# Objections to the Operation for Hydrocele by Injection, in Comparison with That by Incision

**Published:** 1841-08

**Authors:** Thomas Carroll

**Affiliations:** Cincinnati, Ohio


Art. III.—*Objections to the Operation for Hydrocele by Injection, in comparison with that by Incision.* By Thomas Carroll, M. D., of Cincinnati, Ohio.

I believe most medical men in this country are favorable to the operation for Hydrocele by injection, although there are not a few well authenticated facts going to show that this mode of operating has not unfrequently failed, and that it has sometimes been followed by the most disastrous consequences. Failure, indeed, has followed where the operation was performed in the best manner; and evidence is not wanting to prove that death has resulted from it, where little or no blame could be attached to the unfortunate operator. If the tunica vaginalis be healthy, and a certain degree of excitability exist, so that the liquid injected will stimulate just enough to cause the production of that peculiar fluid by which serous surfaces are united, the operation will prove successful, unless some unforeseen circumstance should take place. But where this state of things does not exist, failure will be the result. The following cases will serve to demonstrate this matter better than any train of reasoning which I could lay before the reader:

L. H. of Belmont county, Ohio, aged 60, called on me some time during the summer of 1840, for the purpose of obtaining relief from an enlargement of the scrotum, which had commenced 28 years before. I found the patient in bed, with pain in the loins, fever, and soreness of the tumor, which occasionally existed; but, within the last few weeks, had been more severe and of longer duration, so that the patient was mostly confined to bed.

On examination, I found a hydrocele of the left side, reaching nearly to the knee, being twenty-two inches in its greatest circumference, and sixteen in its least. The prepuce was found on the side of the sac about one-third of its length downwards; the glans penis was more than two inches farther back, yet it could be pressed through the opening of the prepuce when necessary. My patient had never, until within a few days of the time of my visit, submitted himself to the examination of a physician, but was now willing to undergo any operation which would relieve him of his sufferings.

I proceeded to operate by making an incision on the upper part of the sac about six inches long, which reached to the more depending portion. I found immense thickening of the parietes, with enlargement of the cord, epididymis, and testicle, but this latter organ was not so much increased in size as the other tissues. The cord and epididymis were each not less than three-fourths of an inch in diameter. The upper part of the sac had become partially ligamentous, in consequence of the immense appendant weight. Probably the sac with its contents, after the discharge of water, would have weighed three pounds. On examining the inner surface of the sac, I found two ulcers, one on the inferior portion of the cord, and the other on the testicle; these ulcers were each the size of a ten-cent piece, and of a black color. A strip of soft muslin was laid in the wound, with each end emerging from the angles of the incision, the lips of which were approximated by adhesive strips. During the first forty-eight hours fomentations of warm water were constantly applied, after which emollient poultices were used for about two weeks; then the sore was dressed with simple cerate, until the cure was perfected, which took place in a short time. In this case, as in all others which I have witnessed, a very large amount of serum was poured out during the first three days, after which the discharge was greatly lessened, becoming gelatinous, and eventually purulent.

Had this patient been operated on by injection, the result must have been suppuration, if not mortification;—convulsions might have supervened before either of these terminations, which would have closed the sufferings of the patient by death in a most horrible form. The following case will serve to confirm this opinion.

J. B. of Guernsey county, Ohio, called on me sometime during the year 1839, and said that he had a hydrocele of some years standing, and that he had been operated upon twice, once by puncture and once by seton, without having obtained any relief. He stated that the seton gave him much pain, and that it had been withdrawn too soon. This relation made me suspect a peculiarity of constitution which would need much caution, in case of an operation, to prevent violent inflammation. I told him I could cure him if he would submit to an operation, to which he assented; and I accordingly operated by making an incision, about two and a half inches long. A few ounces of water were discharged, and the wound was dressed by laying in it a strip of muslin, and drawing the lips together with adhesive plaster. I now ordered fomentations to be occasionally applied, and the patient was directed to live on bread and water, or on bread and weak tea. These directions were disregarded, the patient stating that he had no suffering, and therefore needed neither fomentations nor starvation. About the end of the second day, I became anxious that the patient should take a cathartic and be bled, as there were symptoms of approaching fever; but I was again disappointed by his obstinacy. No untoward symptoms, however, took place until about the end of the third day. The discharge had now become small in quantity and gluey, and the parts were more swollen, and sore to the touch. General fever now existed, with some restlessness; this condition continued for a short time, when convulsions of the muscles of the extremities came on, and were for a few minutes very severe. I soon arrived, however, and immediately withdrew all the dressings, applied fomentations to the scrotum, and bled the patient largely. The cramps subsided in a few moments, and no further untoward symptoms took place; but contrary to my expectations no suppuration followed within the sac. The external wound healed very soon, and it was hoped that an obliteration of the sac had been produced; this hope, however, was not realized, for a very slow accumulation of fluid took place soon after the healing of the wound.

I told my patient that I could still cure him, if he would follow my directions after another operation; and that if he submitted to an operation by any other physician, the whole nature of his case should be fairly stated, together with the true cause of the failure of the operation by myself.

Sometime in March, 1841, this man submitted to another operation, which was performed by injection. Unfavorable symptoms very soon developed themselves, and it was thought that part of the injection had been thrown into the cellular membrane. However this may have been, I know not, but the patient had violent inflammation, with, I believe, loss of sensation of the side of the body on which the hydrocele was situated; likewise, delirium, cramps, and mortification of the scrotum, which soon closed his sufferings. Now I was of the opinion before the unhappy fate of this individual occurred, that if he should ever be operated upon by injection, a fatal result would follow, whether the cellular tissue were injected or not. The extremely irritable nature of the patient's constitution led me to this conclusion.

About the year 1825, the late Dr. Dickson, of Steubenville,

Ohio, operated on a gentleman for hydrocele by injection. Much pain was experienced at the time, but the patient recovered in a short period from all painful symptoms; it was found, however, that the operation had not succeeded. This gentleman visited Steubenville during the winter of 1825–26, for the purpose of being again operated on by a professional gentleman of that place; but it was discovered that adhesion had taken place immediately around the point where the trocar had entered, in consequence of which, the water had accumulated in such a way that the testicle was pushed downwards, so that the accumulation seemed to be mostly above that organ. This circumstance led to the conclusion, that the trocar could not be inserted into the sac without danger to the cord, and that a larger accumulation must be allowed to take place, before a second operation could be safely performed.

In May, 1826, I was consulted by this gentleman as to the probabilities of relief by a second operation. I advised the operation by incision, which he agreed to, and I operated as in the preceding cases. The patient was directed to live on bread and water, and to use occasionally fomentations and thin poultices to the wound for some days; after which the sore was dressed with simple cerate. A perfect recovery took place in a short time, with a radical cure of the disease.

In the foregoing observations, it has been no part of my purpose to go into a general description of the different operations that have been proposed for the relief of hydrocele; but only so far as experience has taught, to give my *objections* to the operation by *injection*, and in favor of that by *incision.*

